# Social support may not impact physical function outcomes following a tango or walking intervention in people with Parkinson’s disease: an exploratory analysis of a randomized controlled trial

**DOI:** 10.3389/fpsyg.2025.1525172

**Published:** 2025-07-16

**Authors:** D. A. Jehu, J. Bek, M. E. Hackney

**Affiliations:** ^1^Department of Community & Behavioral Health Sciences, Institute of Public and Preventive Health, School of Public Health, Augusta University, Augusta, GA, United States; ^2^School of Psychology, University College Dublin, Dublin, Ireland; ^3^Centre for Motor Control, Faculty of Kinesiology & Physical Education, University of Toronto, Toronto, ON, Canada; ^4^Division of Geriatrics and Gerontology, Department of Medicine, Emory University School of Medicine, Atlanta, GA, United States; ^5^Atlanta VA Center for Visual and Neurocognitive Rehabilitation, Atlanta, GA, United States; ^6^Birmingham/Atlanta VA Geriatric Research Education and Clinical Center, Atlanta, GA, United States

**Keywords:** Parkinson’s disease, neurorehabilitation, social support, dance, exercise, tango

## Abstract

Poor physical function influences social support among people with Parkinson’s disease (PD). Physical function can be improved through exercise, but whether social support influences intervention responsiveness is unclear. This study aimed to (1) examine the influence of 12 weeks of group exercise (tango or walking) on physical function in people with PD who reported low versus high baseline social support, and (2) determine the influence of intervention type on social support effects. This exploratory analysis of an ongoing assessor-blinded randomized controlled trial (1:1) [NCT04122690] included 40 participants with PD (69.24 ± 7.73 years; 26.3% female; Hoehn & Yahr: 2.28 ± 0.58). We used the Multidimensional Scale of Perceived Social Support to categorize participants into high or low support groups. Participants were assessed OFF medication at baseline and 12 weeks using the timed-up-and-go, 360-degree turn, forward, backward, and fast gait speed, 6-min walk, chair stand, and tandem stance tests. Participants were randomized into tango (*n* = 20) or walking (*n* = 20) groups and completed 20 sessions within 12 weeks. Left foot tandem stance trended toward improvement regardless of social support (*p* = 0.06). An interaction among time, support, and intervention indicated that participants in the walking group with low social support improved more on the chair stand test (*p* = 0.03). No adverse intervention-related events occurred. Overall, high and low social support groups improved similarly following tango or walking interventions. Those in the walking group with low support may have benefited more. Therapeutic intervention targeting physical function regardless of social network in people with PD is important.

## Introduction

Parkinson’s disease (PD) can interfere with social functioning, which can impact the overall health of those with PD (Henry et al., 2016). Social functioning can be impacted by several factors, such as age, disease, mobility limitations, and cognitive impairment. Older adults often have a smaller social network and reduced closeness to network members compared to young and middle-aged adults, due to factors such as retirement and bereavement (Cornwell et al., 2008), as well as chronic health problems, sensory impairment, and income instability (National Academies of Sciences, 2020). In particular, mild cognitive impairment is common among people with PD, and those who are aware of their own cognitive decline may avoid social activities, especially highly demanding cognitive activities (Ho et al., 2021; Anderson, 2019) or activities requiring digital literacy (Zhu et al., 2023) due to embarrassment and stigmatization (Zhu et al., 2023). People with PD may also have difficulty recognizing emotions, and such social impairment is associated with greater PD burden (Coundouris et al., 2019). PD also leads to decreased mobility, and those who have mobility challenges tend to report social withdrawal due to a lack of required physical support, stigmatization, and embarrassment (Ahn et al., 2022; Nilsson et al., 2015). Physical function and social support appear to be related, as adults aged 18–64 years with mobility disability who were unemployed and in fair or poor health reported greater loneliness, more isolation, and less satisfaction with social activity (Hall et al., 2022). Given that social support plays a protective role in overall health (Frick et al., 2012), and that people with PD have poorer social functioning (Henry et al., 2016), it is important to understand the impacts of reduced social support in this population.

Social support protects overall health in several ways. Forms of social support can include emotional, informational (e.g., advice leading to a solution to problems), instrumental (e.g., practical help), and companionship support (Ferlander, 2007; Thoits, 2011). A greater number and quality of social ties may increase a sense of belonging to others, self-esteem, and a sense of control over life situations (Thoits, 2011). Stress-buffering effects of social ties may be the mechanism of action explaining better health outcomes with greater social support (Thoits, 2011). Social support offered as facts or recommendations from others–such as where to find less expensive and more convenient goods and services–enables subsequent behavioral changes that make everyday vexing circumstances more efficient (Thoits, 2011). Coping assistance strategies are stress buffers because they dampen situational demands and the person’s emotional reactions to these demands, which can reduce the physical and psychological consequences of the stressor (Thoits, 2011). Social support also buffers the impact of adversity (Thoits, 2011). The social resource model argues that social relationships are embedded within social ties and framed as social resources or social capital, which can demonstrate benefits to physical health outcomes (Song et al., 2021). While social roles for people with PD may change throughout the course of the disease, social support can benefit people with PD by encouraging health-promoting peer social norms, such as adequate educational programs and exercise, and discouraging health-damaging behaviors such as smoking, excessive eating, and drug use (Perepezko et al., 2019). Social ties can also facilitate access to healthcare resources, such as medical referrals, life skills training, education, or support groups (Ferlander, 2007). Therefore, given the importance of social support for health outcomes, examining the impact of different levels of social support on response to interventions is important for those with PD.

Group exercise improves physical function and social connectedness in people with PD (Hackney and Earhart, 2010; McKee and Hackney, 2013). This improved connection may stem from increased socialization among participants and instructors during group exercise (Mays et al., 2021). Thus, clinical trials of group exercise often control for socialization in the design of the intervention when contrasting lifestyle intervention effectiveness with a comparison group (Capili and Anastasi, 2023; McKee and Hackney, 2013; Hackney and Earhart, 2010). However, people with PD who are socially isolated are less physically active (Terracciano et al., 2023) and exhibit poorer physical function than socially connected older adults (Ahn et al., 2022). Therefore, baseline levels of social connectedness may influence the responsiveness of physical function outcomes to intervention. A better understanding of the role of social support in physical function responsiveness to exercise interventions among people with PD could provide insight into more effective treatment strategies.

The purpose of this exploratory analysis of an ongoing randomized controlled trial (RCT) was to examine the influence of 12 weeks of group exercise (tango or walking) on physical function in people with PD who reported low perceived social support compared to those with high perceived social support. As a secondary aim, we explored whether the type of intervention (tango or walking) influenced the impact of social support on physical function outcomes. We hypothesized that people with PD with high social support would exhibit greater improvements in physical function than the low social support group following 12 weeks of either tango or walking exercise. We also hypothesized that we would observe no differences between the tango or walking interventions for the low compared to the high social support groups on physical function.

## Methods

### Study sample, recruitment, and randomization

The sample was drawn from the PAIRED protocol, which is a parallel, assessor-blinded RCT (1:1) that has been registered on clinicaltrials.gov (NCT04122690), and the protocol has previously been published (Hackney et al., 2020). The Consolidated Standards of Reporting Trials (CONSORT) were followed to ensure the quality of reporting (Schulz et al., 2010).

We recruited participants from the Atlanta VA Medical Center Movement Disorders Clinic and the Emory Movement Disorders Clinic, at local PD-related support groups, the Michael J. Fox Foundation FoxFinder web-based registry, educational meetings, and community events. Recruitment for the sample considered in this paper took place between November 2020 and March 2024. All participants provided written informed consent. The Emory IRB and the VA Research and Development committees approved all protocols (Emory IRB112770).

Participants met the following inclusion criteria: (1) a diagnosis of PD (ICD-10 G20) based on established criteria (Hughes et al., 1992) and determined by a board-certified neurologist with training in movement disorders; (2) asymmetric symptoms including at least three of the cardinal signs of PD (rigidity, bradykinesia, tremor, postural instability) and showed clear symptomatic benefit from anti-Parkinsonian medications (Kempster et al., 2007); (3) aged 40 years or older; (4) a Hoehn & Yahr stage I–III; and (5) reported symptoms during their “off” time indicated by score ≥1 on UPDRS-IV item 4.3 (i.e., time spent in the OFF-state). Exclusion criteria included scoring <17 on the Montreal Cognitive Assessment (Litvan et al., 2012) or ≥18 on the Beck Depression Inventory-II (Schrag et al., 2007), or having another neurodegenerative disease.

Study staff assigned participants randomly to an adapted Argentine tango (tango) or supervised group walking (walking) program after baseline assessments. Participants were randomized using the REDCap randomization module, stratified by age and sex (Harris et al., 2009). Investigators and administrative staff, but not instructors, were blinded to treatment assignments. Participants knew their treatment but not whether they were allocated to an experimental or control group.

### Procedure

The testing and training took place at the Atlanta VA North Dekalb Community-Based Outpatient Clinic. We provided transportation for participants who needed it. All participants were tested on physical function at baseline and 12–13 weeks. Participants were tested on all outcome measures at least 12 h after their last dose of anti-Parkinsonian medications (OFF-medication) at a standardized time of day to obtain an accurate assessment of disease severity and evaluate treatment effectiveness. All participants were ON medication during all intervention sessions.

### Participant characteristics

Participants reported demographic characteristics on a health questionnaire (see Table 1). Global cognition was assessed using the Montreal Cognitive Assessment (MoCA; Nasreddine et al., 2005). A Movement Disorders Society-certified rater (blinded to group allocation) evaluated participants on the Movement Disorders Society revision of the Unified Parkinson’s Disease Rating Scale (MDS-UPDRS), parts I-IV (Goetz et al., 2008). Physical activity levels were measured using the Physical Activity Scale for the Elderly (Washburn et al., 1993). Participants rated leisure-time physical activity on a 4-point Likert scale, with lower scores indicating less activity. The Paffenbarger College Alumnus PAQ (PAQ-P) is a measure of the number of kilocalories expended in a week during physical activity and queries daily blocks walked, daily flights of stairs climbed, and recreational activities (including frequency and duration of each episode) as reported on the PASE (Rubenstein et al., 2011). A total Paffenbarger physical activity score (PAS-P) is tabulated by summing the kilocalories expended during blocks walked, stairs climbed, and recreational activities. Scoring assumes all participants are the same weight (60 kg); thus, we divided the PAS-P by 60 kg, resulting in kcal expended per kg of body weight per week.

**Table 1:** Participant characteristics; mean (standard deviation).

Variable	All participants (*n* = 38)	Low support (*n* = 14)		High support (*n* = 24)		Low vs. high support (statistic, *p*-value)
		Tango (*n* = 7)	Walk (*n* = 7)	Tango (*n* = 12)	Walk (*n* = 12)	
Age, years	69.24 (7.73)	65.71 (9.59)	66.57 (6.21)	71.33 (6.21)	70.75 (8.45)	1.92 (0.07)
Female Sex, *n* (%)	10 (26.32)	2 (28.57)	4 (57.14)	3 (25.0)	1 (8.33)	2.91 (0.16)
MDS-UPDRS, part I score	10.84 (6.28)	13.86 (8.23)	11.14 (6.49)	9.08 (4.56)	10.67 (6.51)	−1.17 (0.26)
MDS-UPDRS, part II score	14.70 (9.88)	12.86 (10.29)	19.43 (12.71)	12.82 (7.32)	14.75 (10.25)	−0.64 (0.53)
MDS-UPDRS, part III score	38.37 (14.42)	43.57 (15.89)	39.57 (16.87)	36.42 (9.19)	36.58 (17.20)	−1.00 (0.33)
MDS-UPDRS, part IV score	6.08 (4.12)	6.71 (4.96)	5.71 (3.40)	5.58 (3.12)	6.42 (5.20)	−0.15 (0.88)
Time since PD onset, years	6.91 (5.41)	6.57 (4.65)	6.79 (5.58)	5.08 (4.20)	9.00 (6.61)	0.21 (0.84)
Hoehn & Yahr, score	2.28 (0.58)	2.29 (0.57)	2.36 (0.75)	2.29 (0.40)	2.21 (0.69)	−0.35 (0.73)
MoCA, score	26.37 (3.16)	26.00 (4.08)	27.43 (1.72)	26.25 (2.77)	26.08 (3.78)	−0.52 (0.61)
Education, years	16.53 (2.36)	15.71 (3.15)	16.29 (2.43)	16.50 (2.43)	17.17 (1.80)	0.99 (0.33)
PASE score	120.46 (98.02)	212.20 (172.26)	95.46 (90.09)	115.30 (48.67)	98.29 (89.40)	−0.86 (0.40)
PAS-P, kilocalories expended per week	4017.34 (5717.74)	9749.10 (7979.55)	2841.73 (5897.78)	3741.88 (5419.19)	2460.62 (3515.69)	−1.11 (0.28)
MSPSS baseline	5.93 (1.32)	4.13 (1.68)	5.14 (0.88)	6.66 (0.32)	6.71 (0.31)	5.43 (<0.001)
MSPSS post-training	6.11 (0.90)	5.48 (1.38)	5.68 (0.93)	6.22 (0.84)	6.57 (0.48)	2.31 (0.03)

MoCA, Montreal Cognitive Assessment; MDS-UPDRS, Movement Disorders Society Unified Parkinson Disease Rating Scale; PASE, Physical Activity Scale for the Elderly; PAS-P, Paffenbarger physical activity score, kilocalories expended per week; MSPSS, Multidimensional Scale of Perceived Social Support.

### Stratification by perceived social support

This study operationalized social support using the Multidimensional Scale of Perceived Social Support (MSPSS). The MSPSS is a measure of perceived social support from family, friends, and significant others (Zimet et al., 1990). The MSPSS comprises 12 questions rated on a 7-point Likert scale (1 = very strongly disagree, 7 = very strongly agree), with higher ratings reflecting greater perceived social support. The MSPSS has been used in other studies to assess social support in people with PD (Ghorbani Saeedian et al., 2014; Chen, 2025). The average overall score was used to stratify participants. According to previously defined cutoffs for the MSPSS, a score of 1 to 2.9 is considered low support, a score of 3 to 5 is considered moderate support, and a score from 5.1 to 7 is considered high support (Zimet et al., 1990). Because of our sample size we categorized participants into high or low groups only; the *high social support* group reported “strongly agree” or “very strongly agree” for total perceived social support (equivalent to average scores of 6–7 on the MSPSS), and the *low social support* group reported “mildly agree,” “neutral,” “mildly disagree,” “strongly disagree,” or “very strongly disagree” (equivalent to average scores of 1–5 on the MSPSS).

### Physical function outcome measures

The timed-up-and-go (TUG) is a valid and reliable measure of functional mobility in PD (da Silva et al., 2017), and involves getting up from a chair, walking 3 meters, turning around, walking back, and sitting down (Lee et al., 2020; Ries et al., 2009). Participants completed the TUG as a single task, as well as a dual task while counting backward by 3 from 100.

We measured turning with the 360-degree turn test (Prime et al., 2020). Participants were asked to turn 360 degrees left at a self-determined pace, and we recorded the time to completion. Turning measures are valid and reliable in PD (da Silva et al., 2017).

We measured forward, backward, and fast gait speed across 6.1 meters. We instructed participants to walk at their usual pace during the forward and backward gait conditions, and as fast as they could without running during the fast gait speed condition. The time to completion was recorded in seconds and converted into a gait speed in m/s. These assessments have been sensitive to the effects of PD (Hackney and Earhart, 2009; Hackney and Earhart, 2010). Gait measures are valid and reliable in PD (da Silva et al., 2017; Kocer et al., 2023).

Participants completed the 6-min walk test, which is valid and reliable in PD (Bailo et al., 2024), and were asked to walk as far as they could without running (ATS, 2002). The total distance was measured in meters.

For the chair stand test, participants were asked to stand up and sit down from a chair as many times as possible in 30 s. The number of chair stands was recorded. Sit-to-stand and stand-to-sit measures are valid and reliable in PD (da Silva et al., 2017).

For the tandem test, participants were asked to stand as long as possible with one foot in front of the other for up to 30 s with left or right foot in front. Participants completed two trials, of which the best was used in analyses. The tandem test is valid and reliable in PD (Thomas et al., 2004).

### Intervention design and structure

All intervention participants were asked to complete 20 lessons that were 90 min each in length within 12–13 weeks after baseline assessments. According to the social resource model, social relationships can benefit physical health (Song et al., 2021). Therefore, all participants had a similar exposure to social interactions with the research team. The rate of perceived exertion (RPE) on the Borg scale and heart rate (HR) were taken pre-warm up, post-warm up, and at 15-min, 30-min, and 45-min time points. Participants wore a chest-strapped Polar heart rate monitor during the exercise session, and were monitored with a connected IPad during the sessions. For the Borg, participants were asked to work at a rate of perceived exertion between 4 to 6 on a 10-point scale. They were coached accordingly to ease up or exert themselves more if the values did not fall within this range. If participants missed a training session, they were contacted and invited to make up for the missed session if they were willing to do so.

#### Tango program

Participants were paired with a neurologically healthy partner (trained staff, university student, caregiver, or friend). The instructor and several trained assistants monitored all participants. Class sizes consisted of 6 or fewer pairs of participants plus their partners to maximize safety. Participants danced leading and following roles and rotated to new partners every 15 min to enhance motor learning and social interaction. Tango was intended to engage participants in developing an understanding of the temporal relationship of movement to music and connecting previously learned and novel step elements. The dance classes included 15-min practica where participants learned previous steps, 25-min standing warmup, 45-min partnered and rhythmic enhancing exercises, and 5-min cooldown. All instructors were trained on motor impairments, fall detection and prevention, and the American Council on Exercise-certified training on adapted tango methods by one of the authors (MEH). In terms of social interaction, participants had the opportunity to speak one-on-one with their dance partner, who changed every 15 min, as well as the instructor.

#### Walking program

Participants allocated to walking received the equivalent dose, volume, frequency, and intensity of exercise as the tango group. Participants reported to the same facility and received equal contact and monitoring from study staff. Walking sessions consisted of a 20-min warm-up, with tips for safe walking mechanics; 45 min of walking outside with breaks as needed; and 5 min of stretching and balance exercises (Wells et al., 2023). Walking for exercise expends 4.28 metabolic equivalents (Knaggs et al., 2011), similar to 4.3 metabolic equivalents spent on tango dancing (Santos-Silva et al., 2021). Participants started slowly and worked their way up to 60–65% of their heart rate maximum and set clear goals regarding speed and length of walking. Participants also used an application on their phones to record the distance walked and the number of steps taken. The outdoor overground walking took place in a safe, non-cluttered environment in groups, with staff and assistants. During poor weather or if preferred, participants walked indoors. In terms of social interaction, participants had the opportunity to speak with a group of participants who walked at a similar pace, as well as the instructor.

### Analysis

Demographic characteristics at baseline were compared between high and low social support groups using independent t-tests (except for sex, which was analyzed using Fisher’s exact test). Baseline measures of physical function were compared between the high and low social support groups using independent t-tests.

The primary analysis examined the effects of time (baseline, post-intervention) and social support (high, low) on each of the physical function outcomes using linear mixed-effects models (LMM). The models included fixed effects of Time and Support as interaction terms and random intercepts for participants.

A secondary analysis examined whether the specific intervention received might influence outcomes. The factor Intervention group (walking, tango) was added to each model as an additional interaction term (Time*Support*Intervention). Models with and without this factor included were compared using likelihood ratio tests, and the best-fitting model for each outcome is reported below.

Finally, we examined the effects of interventions on social support levels, using LMM to analyze main effects and interactions of Time and Intervention on the total MSPSS score. Statistical analyses were conducted in R (R Core Team, 2023) using the packages “lme4” (Bates et al., 2015) and “emmeans” (Lenth et al., 2024). The alpha was set at <0.05.

## Results

### Participant characteristics

**Figure 1:**
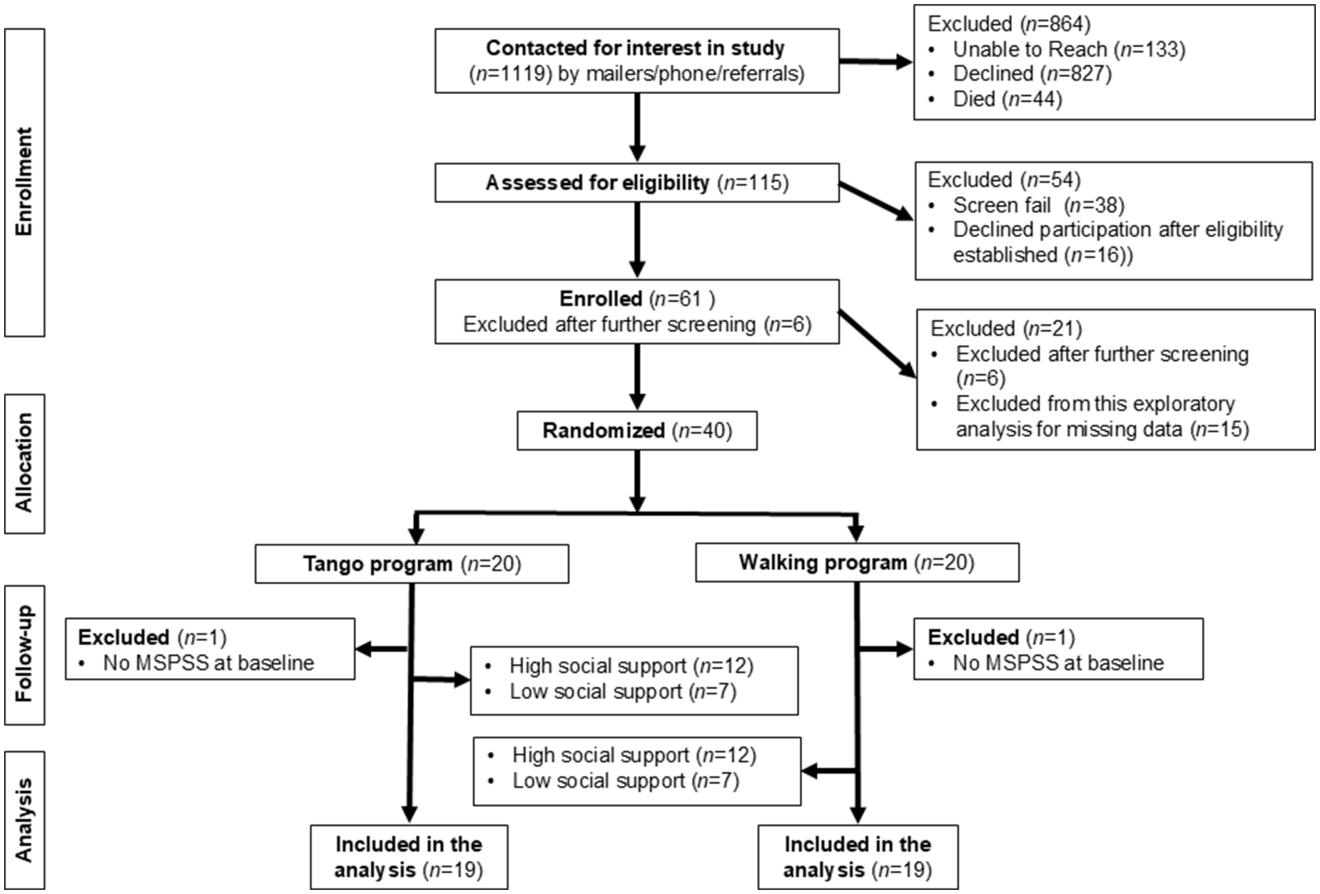
Study flow diagram and procedure for the PAIRED trial.

At the time of this exploratory analysis, 1,119 people with PD were initially contacted by phone or mail advertising. Of these, 827 declined to participate, 133 did not respond and 44 died. Screening of 115 individuals was completed, of which 38 did not meet the eligibility criteria, and 16 dropped out after initial screening and before baseline assessments. Sixty-one participants consented to participate in the study, 6 of whom did not complete baseline assessments. Therefore, at the time of this analysis, 55 participants were randomized into tango or walking. However, 17 of these participants had incomplete data, including missing MSPSS scores, not remaining in the study long enough to complete 12–13 weeks of training, or were excluded for other reasons. As such, we include in these analyses 19 participants in each arm with complete data (Figure 1). Exercise adherence was high for both walking (95.5 ± 19.9%) and tango (97.2 ± 6.88%) interventions. Based on the total MSPSS score, the final analysis included 24 participants with high perceived social support (12 in the tango group, 12 in the walking group) and 14 with low perceived social support (7 in each group). There were no adverse events reported as a result of either intervention. Across the two intervention groups, no significant differences were found between participants with high vs. low social support on any of the demographic characteristics (Table 1) or physical function measures (Table 2) at baseline. Levels of social support (MSPSS scores) did not change significantly from baseline to post-intervention, and there was no interaction between time and group on MSPSS score.

**Table 2:** Physical outcome measures at baseline in participants with low or high social support; mean (standard deviation).

Variable	Low support (*n* = 14)				High support (*n* = 24)				Baseline comparison low vs. high support (t, *p*)
	Baseline		Post-training		Baseline		Post-training		
	Tango	Walk	Tango	Walk	Tango	Walk	Tango	Walk	
TUG single, s	12.79 (10.00)	14.20 (6.00)	15.20 (14.51)	11.78 (5.19)	12.50 (6.91)	10.93 (3.35)	10.25 (9.06)	11.31 (3.66)	0.07 (0.95)
TUG dual, s	15.51 (9.21)	21.22 (13.31)	17.89 (9.26)	21.51 (19.14)	23.94 (29.86)	23.14 (27.74)	15.15 (9.04)	15.13 (6.04)	0.48 (0.63)
360 turn left, s	3.79 (3.49)	5.43 (2.31)	4.14 (1.97)	5.06 (3.61)	6.28 (7.27)	5.14 (3.66)	3.63 (3.17)	4.80 (2.27)	−0.01 (0.99)
360 turn right, s	3.95 (3.40)	6.68 (5.45)	13.80 (35.12)	6.56 (7.04)	10.23 (21.68)	5.32 (3.25)	3.59 (2.92)	5.44 (3.22)	0.92 (0.36)
Gait forward, m/s	1.09 (0.40)	1.01 (0.20)	1.08 (0.36)	1.02 (0.35)	1.02 (0.38)	1.05 (0.33)	1.22 (0.44)	1.02 (0.30)	0.19 (0.85)
Gait backward, m/s	0.67 (0.37)	0.50 (0.15)	0.63 (0.23)	0.73 (0.44)	0.64 (0.27)	0.65 (0.34)	0.73 (0.41)	0.55 (0.28)	0.87 (0.39)
Gait fast, m/s	1.56 (0.59)	1.29 (0.27)	1.37 (0.52)	1.36 (0.83)	1.38 (0.43)	1.55 (0.51)	1.79 (0.59)	1.44 (0.63)	−0.20 (0.84)
6-min walk test, m	425.20 (134.46)	313.59 (118.19)	343.23 (141.00)	308.36 (155.44)	348.24 (146.66)	308.20 (152.26)	381.69 (173.60)	364.90 (145.92)	−1.20 (0.25)
Chair stand test, total #	14.00 (7.85)	8.14 (3.67)	11.67 (3.63)	11.17 (2.95)	11.67 (4.08)	11.55 (2.42)	12.71 (6.85)	12.13 (3.72)	0.46 (0.65)
Tandem left foot, s	18.12 (14.99)	20.76 (11.68)	17.68 (11.64)	16.24 (12.93)	21.20 (13.03)	20.75 (10.92)	23.40 (10.22)	24.24 (10.92)	−0.95 (0.35)
Tandem right foot, s	13.69 (15.32)	22.89 (9.22)	21.40 (9.46)	20.65 (11.89)	22.05 (12.00)	20.05 (11.45)	23.48 (9.88)	21.53 (12.15)	0.43 (0.67)

TUG, Timed Up and Go; TUG dual uses the cognitive dual task. # in this case means “total number of chair stands.

### The influence of social support on physical function following tango or walking interventions

**Figure 2:**
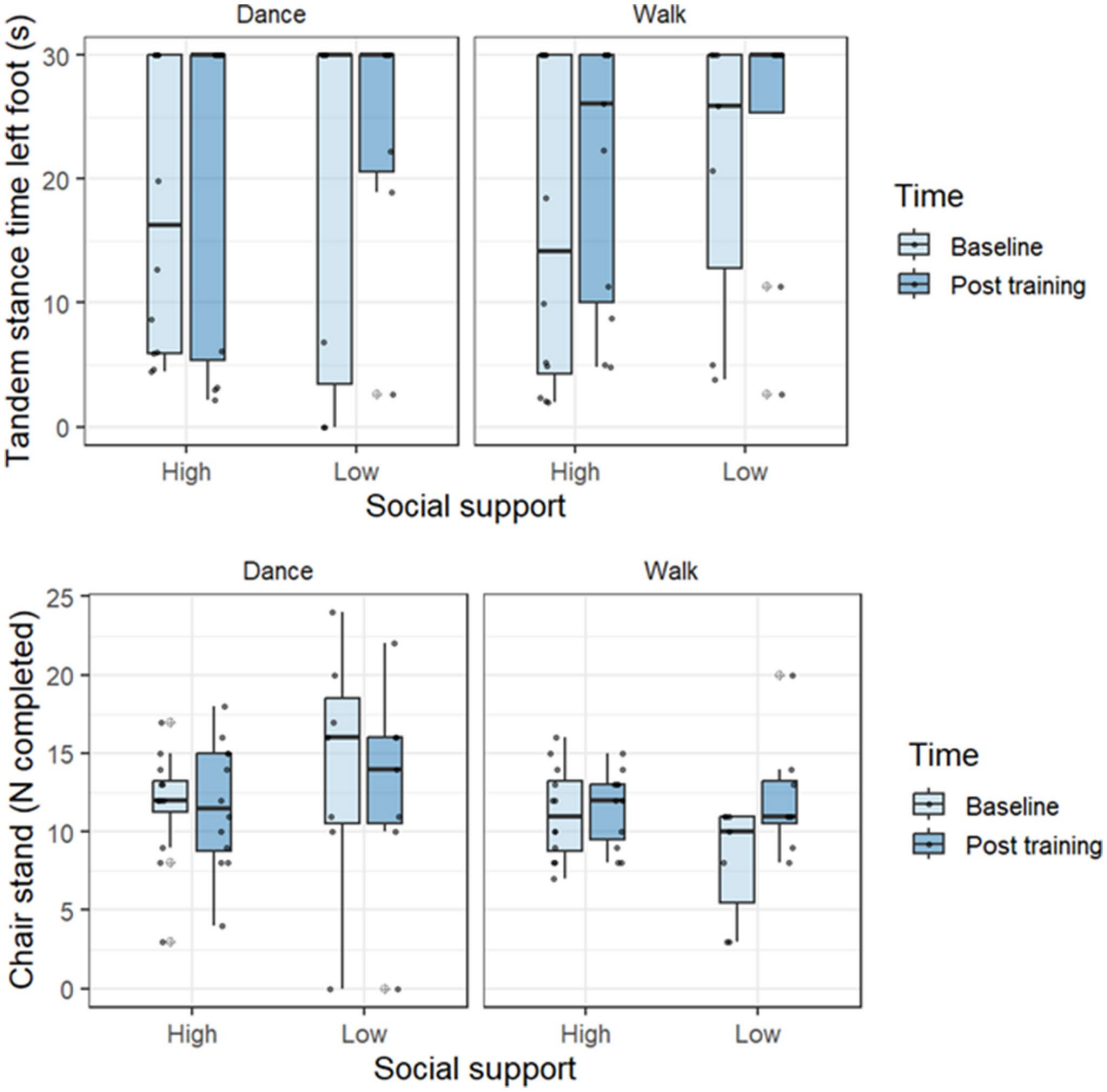
Performance on the tandem stance test and chair stand test at baseline and post-training in participants with high and low perceived social support. Boxes show medians with interquartile ranges; dots represent individual datapoints. Tandem stance time showed a trend toward increasing regardless of social support level, while the number of chair stands completed increased significantly in participants with low social support in the walking intervention only.

Full model outputs for the LMM are provided in Supplementary material 1. In the primary analysis, a non-significant trend indicating an effect of time was found for the tandem stance test with the left foot forward [*b* = 4.33, *SE* = 2.27, *t*(38.23) = 1.90, *p* = 0.06; Figure 2]. A trend indicating an effect of support was found for the six-minute walk test [*b* = 280.57, *SE* = 145.16, *t*(63.60) = 1.93, *p* = 0.06]. No other significant effects were found on any of the outcome measures.

Secondary analyses found that including the intervention group significantly improved the fit of the model for the chair stand test, with a significant interaction between time, support, and intervention group [*b* = 3.50, SE = 1.53, *t*(37.48) = 2.28, *p* = 0.03; Figure 2]. Comparison of estimated marginal means revealed a significant improvement in participants with low social support receiving the walking intervention [adjusted M = −3.07, SE = 0.83, *t*(43.6) = −3.42, *p* = 0.001], but not in participants with high support receiving the walking intervention, or participants with either low or high support receiving the tango intervention (*p_s_* > 0.1). Adding the intervention group did not significantly improve the model fit for any of the other outcome measures.

## Discussion

To our knowledge, this is the first study to examine whether baseline social support levels influence potential benefits in physical function following therapeutic intervention among people with PD. We found that both high and low social support groups similarly improved in physical function following group exercise (tango or walking). Secondary analyses examining the potential influence of intervention type revealed that people with PD who had low baseline social support and received a walking intervention demonstrated more improvement in the number of chair stands than those who received tango in the high or low social support groups or those in the walking program with high social support.

Contrary to our hypothesis, we found that participants with either high or low social support exhibited largely similar physical function improvements. Across high and low social support groups, participants showed a trend toward improvement in the left tandem stance test. Additionally, both groups showed a clinically meaningful improvement in fast gait speed (>0.1 m/s; Palombaro et al., 2006) following the 12-week intervention. This finding is encouraging, as the intervention likely not only challenged physical function but also fostered social integration, social cohesion, and a sense of belonging (Hawkins et al., 2016) among participants and staff. This social cohesion may have been particularly important for those with low social support, as it could have provided them with the social resources they needed, greater resilience, and a greater propensity for improved physical function outcomes, which aligns with the social resource model (Song et al., 2021). However, it is possible that the low social support group did not socially connect during the intervention, and the improvements in physical function could be solely due to exercise. Nevertheless, our findings highlight the importance of therapeutic intervention in improving physical function regardless of social support in people with PD.

Our exploratory analysis of an ongoing RCT only found an improvement in one outcome measure (chair stand test) for participants in the low social support group receiving the walking intervention compared to the high social support group receiving the walking intervention or those with high or low social support receiving the tango intervention. While the dose, volume, frequency, intensity, and adherence of exercise were similar in the walking and tango interventions and led by the same intervention staff, differences in the level of social engagement or the specificity of training may explain these findings. For example, the topics of discussion during the tango program were likely centered on problem-solving when learning new tango moves, which may have increased cognitive demands for people with PD, especially those with concomitant mild cognitive impairment, as they have shown to have reduced attention capacity (Saunders and Summers, 2011). In contrast, the walking program likely involved socially engaging, participant-driven conversations. These differences in conversational content and social dynamics may explain the walking program’s superior improvement in physical function. We did not record the conversations, so this explanation remains speculative. Social engagement is a major motivator for older adults to engage in physical activity (Spiteri et al., 2019). Alternatively, the walking program may have more closely mimicked the biomechanical demands of the chair stand test, leading to greater improvements. Walking involves repetitive activation of lower body muscles (e.g., quadriceps, hamstrings, and glutes) in a rhythmic, weight-bearing manner, similar to the sit-to-stand motion, which requires concentric and eccentric contractions of these muscle groups. In contrast, tango dancing, while engaging lower body muscles, emphasizes complex, multidirectional movements (e.g., pivots, steps, and turns) that may not directly translate to the repetitive, linear motion of the chair stand test. The principle of training specificity suggests that exercises closely aligned with test movements yield greater improvements (Fleck and Kraemer, 2014). Nevertheless, the improvements in the number of chair stands were only observed in the walking group with low social support, and not high social support; thus, the specificity of training may not fully account for the different results. In sum, social connectedness and/or the type of exercise may have contributed to greater improvements in the number of chair stands in the participants with PD with low baseline social support in the walking program, compared to those with high baseline social support in the walking program or participants in the tango program.

Researchers have questioned why some participants respond to interventions while others do not. Prospective cohort data from people with PD document a robust predictive relationship between greater social support and better health outcomes (Terracciano et al., 2023; Ahn et al., 2022). Prospective longitudinal studies have demonstrated that both structural aspects (e.g., large social network size and cohesion) and functional aspects (e.g., high perceived emotional support) of social support are associated with better physical function among older adults with chronic kidney disease (Slaven et al., 2021). Social support has a robust relationship with improved health through projected security of surviving future health crises, reinforced positive health habits (e.g., healthy eating, exercise), and improved health through social ties (e.g., dampened stress; Ross and Mirowsky, 2002). Nonetheless, our preliminary findings provide evidence that low baseline social support may contribute to greater responsiveness to a walking intervention among people with PD.

Current research demonstrates that people with PD with mobility limitations are at risk of low social connectedness and tend to have poorer health outcomes (Ahn et al., 2022). However, our findings highlight that people with PD who have low social support exhibit similar improvements in physical function following an intervention as those with high social support. Our findings emphasize the importance of therapeutic intervention for people with PD who have varying levels of social support.

Since the present study is the first exploratory analysis examining the role of social support in exercise intervention outcomes for people with PD, larger and longitudinal trials are needed. We defined the level of social support based on the total MSPSS score, but it is unclear whether other aspects of social support not captured by this measure, such as the existence or quantity of social relationships, their formal structure (e.g., density or reciprocity), or the actual content of these relationships (e.g., positive or negative), drive differences in responsiveness of physical function measures to exercise. Future research could consider using scales that measure social networks, such as the Lubben Social Network Scale (Lubben, 1988). The average MSPSS score in the low social support group was 4.6/7 points, and all participants had the means to participate in this study. Possibly, those with very low social support may not have the necessary resources, such as time outside of work, to participate in an intervention. The interventions may have directly improved social network and physical function, or it is possible that the intervention had a positive effect on physiological, cognitive, or psychological function as well as symptoms of PD, which may also explain improvements in the high and low social support groups. Social support and lower physical function may also be correlated, and sometimes those who have the most room for improvement have the biggest gains after physical rehabilitation. Future work could consider examining the proportion of participants in trials who are recruited and retained who have low compared to high baseline social support. Future studies should also examine more objective measures of social inequalities that may influence social support, such as low socioeconomic status or other social determinants of health.

## Conclusion

This exploratory analysis of an ongoing RCT found that people with PD who have high or low baseline social support similarly improved in physical function following a 12-week tango or walking intervention. These results may have implications for clinical care management and lifestyle interventions in people with PD. Given the broader relationship between social network and future health outcomes, clinicians should still monitor people with PD who have low social support, even if they respond similarly to intervention.

## Data Availability

The raw data supporting the conclusions of this article will be made available by the authors, without undue reservation.
