# Supplementary material for: Social support may not impact physical function outcomes following a tango or walking intervention in people with Parkinson’s disease: an exploratory analysis of a randomized controlled trial
Source: Front Psychol. 2025 Jul 16;16:1525172. doi: 10.3389/fpsyg.2025.1525172 (PMC12307284; doi:10.3389/fpsyg.2025.1525172)
Supplement: Supplementary file 1 [file Data_Sheet_1.pdf]

			TUG_single			
Predictors	*Estimates*	*std. Error*	*CI*	*Statistic*	*p*	*df*
(Intercept)	13.36	1.58	10.21 – 16.52	8.44	**<0.001**	68
Time [Post training]	-1.92	1.13	-4.16 – 0.33	-1.7	0.094	68
Social support baseline [Low]	0.22	2.46	-4.69 – 5.12	0.09	0.93	68
Time [Post training] × Social support baseline [Low]	-0.46	1.81	-4.07 – 3.16	-0.25	0.803	68
**Random Effects**						
σ^2	14.12					
$\tau_{00\ ID}$	46.52					
ICC	0.77					
N_{ID}	37					
Observations	74					
Marginal R^2 / Conditional R^2	0.018 / 0.771					
AIC	492.93					
BIC	506.75					

	TUG_dual					
Predictors	*Estimates*	*std. Error*	*CI*	*Statistic*	*p*	*df*
(Intercept)	19.76	3.73	12.32 – 27.20	5.3	**<0.001**	68
Time [Post training]	3.49	3.72	-3.93 – 10.92	0.94	0.351	68
Social support baseline [Low]	-1.36	5.99	-13.31 – 10.58	-0.23	0.821	68
Time [Post training] × Social support baseline [Low]	-6.33	5.98	-18.26 – 5.60	-1.06	0.293	68
**Random Effects**						
σ^2	154.45					
$\tau_{00\ ID}$	170.1					
ICC	0.52					
N_{ID}	37					
Observations	74					
Marginal R^2 / Conditional R^2	0.023 / 0.535					
AIC	638.02					
BIC	651.84					

	Turn_360_left					
Predictors	*Estimates*	*std. Error*	*CI*	*Statistic*	*p*	*df*
(Intercept)	4.63	0.78	3.08 – 6.19	5.93	**<0.001**	70
Time [Post training]	1.05	0.76	-0.47 – 2.57	1.38	0.172	70
Social support baseline [Low]	-0.08	1.27	-2.61 – 2.45	-0.06	0.95	70
Time [Post training] × Social support baseline [Low]	-1.28	1.24	-3.75 – 1.20	-1.03	0.307	70
**Random Effects**						
σ^2	6.77					
$\tau_{00\ ID}$	8.1					
ICC	0.54					
N_{ID}	38					
Observations	76					
Marginal R^2 / Conditional R^2	0.020 / 0.554					
AIC	419.47					
BIC	433.46					

	Turn_360_right					
Predictors	*Estimates*	*std. Error*	*CI*	*Statistic*	*p*	*df*
(Intercept)	9.7	3.17	3.37 – 16.03	3.06	**0.003**	70
Time [Post training]	-2.54	1.7	-5.93 – 0.84	-1.5	0.138	70
Social support baseline [Low]	-3.56	4.74	-13.02 – 5.89	-0.75	0.455	70
Time [Post training] × Social support baseline [Low]	2.09	2.77	-3.42 – 7.61	0.76	0.451	70
**Random Effects**						
σ^2	33.4					
$\tau_{00\ ID}$	233.68					
ICC	0.87					
N_{ID}	38					
Observations	76					
Marginal R^2 / Conditional R^2	0.010 / 0.876					
AIC	597.22					
BIC	611.21					

	gaitfwd_speed					
Predictors	*Estimates*	*std. Error*	*CI*	*Statistic*	*p*	*df*
(Intercept)	1.06	0.07	0.92 – 1.20	15.5	**<0.001**	70
Time [Post training]	0	0.07	-0.14 – 0.14	-0.04	0.968	70
Social support baseline [Low]	-0.03	0.11	-0.26 – 0.19	-0.31	0.761	70
Time [Post training] × Social support baseline [Low]	0.05	0.12	-0.18 – 0.28	0.48	0.636	70
**Random Effects**						
σ^2	0.06					
$\tau_{00\ ID}$	0.06					
ICC	0.49					
N_{ID}	38					
Observations	76					
Marginal R^2 / Conditional R^2	0.002 / 0.487					
AIC	52.12					
BIC	66.11					

	gaitbwd_speed					
Predictors	*Estimates*	*std. Error*	*CI*	*Statistic*	*p*	*df*
(Intercept)	0.68	0.06	0.56 – 0.81	10.66	**<0.001**	68
Time [Post training]	-0.02	0.06	-0.13 – 0.10	-0.32	0.748	68
Social support baseline [Low]	-0.11	0.1	-0.31 – 0.09	-1.08	0.282	68
Time [Post training] × Social support baseline [Low]	0.05	0.09	-0.13 – 0.24	0.55	0.582	68
**Random Effects**						
σ^2	0.04					
$\tau_{00\ ID}$	0.06					
ICC	0.62					
N_{ID}	37					
Observations	74					
Marginal R^2 / Conditional R^2	0.019 / 0.624					
AIC	31.4					
BIC	45.22					

	gaitfast_speed					
Predictors	*Estimates*	*std. Error*	*CI*	*Statistic*	*p*	*df*
(Intercept)	1.37	0.11	1.14 – 1.59	12.06	**<0.001**	70
Time [Post training]	0.15	0.11	-0.08 – 0.37	1.31	0.195	70
Social support baseline [Low]	0.06	0.18	-0.31 – 0.42	0.3	0.764	70
Time [Post training] × Social support baseline [Low]	-0.05	0.18	-0.42 – 0.31	-0.28	0.782	70
**Random Effects**						
σ^2	0.15					
$\tau_{00\ ID}$	0.17					
ICC	0.53					
N_{ID}	38					
Observations	76					
Marginal R^2 / Conditional R^2	0.014 / 0.535					
AIC	126.83					
BIC	140.81					

	Six_min_walk_ft					
Predictors	*Estimates*	*std. Error*	*CI*	*Statistic*	*p*	*df*
(Intercept)	1015.29	94.69	826.34 – 1204.24	10.72	**<0.001**	68
Time [Post training]	31.74	61.82	-91.62 – 155.10	0.51	0.609	68
Social support baseline [Low]	280.57	145.16	-9.10 – 570.23	1.93	0.057	68
Time [Post training] × Social support baseline [Low]	-61	99.43	-259.41 – 137.41	-0.61	0.542	68
**Random Effects**						
σ^2	42464.72					
$\tau_{00\ ID}$	177665.71					
ICC	0.81					
N_{ID}	37					
Observations	74					
Marginal R^2 / Conditional R^2	0.065 / 0.820					
AIC	1093.36					
BIC	1107.18					

	Tandem_L					
Predictors	*Estimates*	*std. Error*	*CI*	*Statistic*	*p*	*df*
(Intercept)	16.97	2.33	12.33 – 21.62	7.28	**<0.001**	70
Time [Post training]	4.33	2.27	-0.21 – 8.87	1.9	0.061	70
Social support baseline [Low]	2.44	3.79	-5.11 – 9.99	0.64	0.521	70
Time [Post training] × Social support baseline [Low]	-0.38	3.7	-7.77 – 7.01	-0.1	0.918	70
**Random Effects**						
σ^2	60.27					
$\tau_{00\ ID}$	72.16					
ICC	0.54					
N_{ID}	38					
Observations	76					
Marginal R^2 / Conditional R^2	0.042 / 0.564					
AIC	585.63					
BIC	599.62					

	Tandem_R					
Predictors	*Estimates*	*std. Error*	*CI*	*Statistic*	*p*	*df*
(Intercept)	20.94	2.25	16.45 – 25.42	9.31	**<0.001**	70
Time [Post training]	0.29	2.38	-4.46 – 5.05	0.12	0.902	70
Social support baseline [Low]	-2.5	3.67	-9.82 – 4.82	-0.68	0.499	70
Time [Post training] × Social support baseline [Low]	3.49	3.88	-4.24 – 11.23	0.9	0.371	70
**Random Effects**						
σ^2	66.24					
$\tau_{00\ ID}$	56.27					
ICC	0.46					
N_{ID}	38					
Observations	76					
Marginal R^2 / Conditional R^2	0.012 / 0.466					
AIC	584.1					
BIC	598.08					

	bpst_prod					
Predictors	*Estimates*	*std. Error*	*CI*	*Statistic*	*p*	*df*
(Intercept)	18.68	2.16	14.37 – 22.99	8.65	**<0.001**	70
Time [Post training]	-0.99	2.4	-5.78 – 3.79	-0.41	0.68	70
Social support baseline [Low]	-0.13	3.54	-7.18 – 6.92	-0.04	0.971	70
Time [Post training] × Social support baseline [Low]	1.32	3.9	-6.46 – 9.11	0.34	0.736	70
**Random Effects**						
σ^2	67.19					
$\tau_{00\ ID}$	45.6					
ICC	0.4					
N_{ID}	38					
Observations	76					
Marginal R^2 / Conditional R^2	0.002 / 0.406					
AIC	580.03					
BIC	594.02					

	Chair_stand					
Predictors	*Estimates*	*std. Error*	*CI*	*Statistic*	*p*	*df*
(Intercept)	11.67	1.21	9.26 – 14.08	9.66	**<0.001**	66
Treatment group [Walk]	-1.23	1.65	-4.53 – 2.07	-0.75	0.459	66
Time [Post training]	0	0.65	-1.30 – 1.30	0	1	66
Social support baseline [Low]	2.33	1.99	-1.64 – 6.31	1.17	0.245	66
Treatment group [Walk] × Time [Post training]	0.86	0.94	-1.02 – 2.74	0.92	0.363	66
Treatment group [Walk] × Social support baseline [Low]	-3.37	2.56	-8.48 – 1.73	-1.32	0.192	66
Time [Post training] × Social support baseline [Low]	-1.29	1.07	-3.43 – 0.86	-1.2	0.236	66
(Treatment group [Walk] × Time [Post training]) × Social support baseline [Low]	3.5	1.53	0.44 – 6.55	2.28	**0.026**	66
**Random Effects**						
σ^2	2.55					
$\tau_{00\ ID}$	14.94					
ICC	0.85					
N_{ID}	38					
Observations	76					
Marginal R^2 / Conditional R^2	0.075 / 0.865					
AIC	403.54					
BIC	426.84					
